# Interprofessional Communication of Clinicians Using a Mobile Phone App: A Randomized Crossover Trial Using Simulated Patients

**DOI:** 10.2196/jmir.4854

**Published:** 2016-04-06

**Authors:** Bhavesh Patel, Maximilian Johnston, Natalie Cookson, Dominic King, Sonal Arora, Ara Darzi

**Affiliations:** ^1^ Department of Surgery and Cancer St Mary's Campus Imperial College London London United Kingdom; ^2^ Imperial Patient Safety Translational Research Centre Department of Surgery and Cancer Imperial College London London United Kingdom; ^3^ Academic Surgical Unit St Marys Hospital Imperial College Healthcare NHS Trust London United Kingdom; ^4^ Google DeepMind London United Kingdom

**Keywords:** communication, mobile phone, pager, applications, apps, escalation of care, simulation

## Abstract

**Background:**

Most hospitals use paging systems as the principal communication system, despite general dissatisfaction by end users. To this end, we developed an app-based communication system (called Hark) to facilitate and improve the quality of interpersonal communication.

**Objective:**

The objectives of our study were (1) to assess the quality of information transfer using pager- and app-based (Hark) communication systems, (2) to determine whether using mobile phone apps for escalation of care results in additional delays in communication, and (3) to determine how end users perceive mobile phone apps as an alternative to pagers.

**Methods:**

We recruited junior (postgraduate year 1 and 2) doctors and nurses from a range of specialties and randomly assigned them to 2 groups who used either a pager device or the mobile phone-based Hark app. We asked nurses to hand off simulated patients while doctors were asked to receive handoff information using these devices. The quality of information transfer, time taken to respond to messages, and users’ satisfaction with each device was recorded. Each participant used both devices with a 2-week washout period in between uses.

**Results:**

We recruited 22 participants (13 nurses, 9 doctors). The quality of the referrals made by nurses was significantly better when using Hark (Hark median 118, range 100–121 versus pager median 77, range 39–104; *P*=.001). Doctors responded to messages using Hark more quickly than when responding to pagers, although this difference was not statistically significant (Hark mean 86.6 seconds, SD 96.2 versus pager mean 136.5 seconds, SD 201.0; *P*=.12). Users rated Hark as significantly better on 11 of the 18 criteria of an information transfer device (*P*<.05) These included “enhances interprofessional efficiency,” “results in less disturbance,” “performed desired functions reliably,” and “allows me to clearly transfer information.”

**Conclusions:**

Hark improved the quality of transfer of information about simulated patients and was rated by users as more effective and efficient, and less distracting than pagers. Using this device did not result in delay in patient care.

## Introduction

Failures in communication can have serious and damaging implications for patient safety [1,2]. Data published by The Joint Commission in the United States has consistently highlighted errors in communication as the most common root cause of sentinel events, with handoff errors in particular being implicated in as many as 80% of all adverse events [3-6]. As such, there is a need for health systems to prioritize the improvement of communication skills and modalities within acute health care settings.

In hospitals worldwide, the most widely used communication system continues to be the 1-way pager device, first introduced over 50 years ago [7]. Despite its widespread adoption, users of pagers report the devices to be highly disruptive and inefficient [8-10]. In most paging systems, a member of staff sends an alert to a colleague requesting to be called back. The alert is received as a loud sound with an extension number to be called shown on the pager’s display. As the only information displayed by the pager is a number to be called, pagers do not effectively allow the urgency of a message to conveyed. Furthermore, information cannot be transferred until the receiver returns the call. Delays in response to a page can cause frustration, prompting additional pages [11]. The frequent interruptions caused by repeated pages alongside ineffective information transfer lead directly to adverse events and safety events [12,13]. More generally, frontline clinicians report that pagers have a negative impact on communication, quality of work, and efficiency [11].

The negative sentiment toward pagers in parallel with advances in communication technologies has encouraged many hospitals to explore alternative solutions to pager systems. Such solutions include 2-way alphanumeric pagers, secure short message service (SMS) text messaging platforms, and task management systems [14-17]. While an ideal communication tool would enable bidirectional, rapid, secure, and nondisruptive transmission of content-rich messages, existing systems have different limitations that fall short of this ideal [18].

As a result, the uptake of the new technologies in the health care setting is not yet widespread. Rogers proposed that new innovations diffuse through five distinct sections of consumer in the process of widespread adoption: innovators, early adopters, early majority, late majority, and laggards. Health care organizations have invested heavily in older communication systems that are still functional and have been slow to transition to emergent technologies. New technologies have therefore not moved beyond the innovators and early adopters [19,20]. In contrast, in hospitals where pagers remain the principal method of communication, health professionals are increasingly taking things into their own hands and using personal mobile phones to communicate with colleagues. This can be through a combination of voice calls, SMS messages, and increasingly messaging platforms such as WhatsApp (WhatsApp Inc, Mountain View, CA, USA) [21,22].

Previous systematic reviews of communication systems have demonstrated improvements in clinicians’ perception of communication when using communication systems other than pagers. [14] A more recent randomized controlled trial highlighted the greater employee satisfaction when using mobile phones for intrateam communication [15]. Mobile phones, therefore, present a significant opportunity for advances in interprofessional communication. Clinicians recognize the advantages that mobile phones offer over paging devices, specifically increased accessibility, clearer communication, and the ability to triage messages based on urgency [23,24].

To address the shortcomings of existing communication systems and take advantage of widespread access to mobile phone devices, an app-based communication system (ABCS) called Hark has been developed by Imperial College London, London, United Kingdom. Hark runs over mobile phones and tablet devices to support clinical task management. In doing so it builds on the specifications we defined to reduce adverse events associated with clinical handoff, prioritization of clinical tasks, failure to rescue, and escalation of care [3,11,25,26].

Hark was designed and developed by a multidisciplinary team of researchers, clinicians, technologists, and software developers according to the Imperial clarify, design, and evaluate approach to the development of digital health (mHealth) solutions [27]. It was developed building on extensive feedback from focus group discussions with multiple stakeholders (including nursing staff, doctors, and hospital managers) to ensure that all features of the app are tailored toward end users [11].

Through its design, Hark aims to address the shortcomings of the pager and the negative perceptions of users specifically around the time taken to respond to communication episodes and the quality of information transferred through the device [10,11,25]. This study aimed to assess the feasibility of using Hark in the clinical environment by determining whether the identified shortcomings had been addressed and whether using an alternative communication system would result in additional delays to patient care.

The aims of this study were (1) to assess the quality of information transfer using pager-based and app-based communication systems, (2) to determine whether using a mobile phone app for escalation of care would result in additional delays in communication, and (3) to determine how end users perceive mobile phone apps as an alternative means of communication to pagers.

## Methods

### Participants

We approached doctors and nurses working at a 500-bed tertiary hospital for participation in the study. Participants were recruited against the following criteria. Inclusion criteria were (1) being a doctor or nurse working in the hospital and (2) working in a specialty that serves inpatients. Exclusion criteria were (1) being a medical or nursing student, (2) being a nurse not directly involved with the routine care of inpatients (eg, operating room staff, scrub nurses, (3) being agency (temporary) staff, and (4) being computer illiterate. Before recruitment, we deemed a sample size of a minimum of 40 data points to be sufficient on the basis of previously published literature involving simulation in the methodology [28-32].

### Study Design

**Figure 1:**
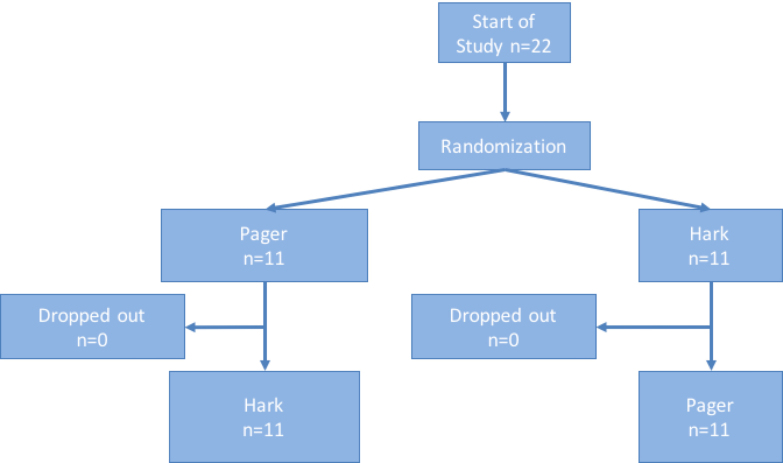
Design of the study.

This study used a randomized crossover design. Participants were randomly assigned into 1 of 2 groups according to a random number generator. We asked one group to use a conventional pager device to transfer information and the other to use Hark. Once participants completed the task, they underwent a 2-week washout period before being asked to perform similar tasks using the other device. By using such a study design, we obtained a greater number of data points using a smaller sample size. Furthermore, as all participants used both devices, they acted as their own internal control to minimize sampling bias between the groups. Figure 1 illustrates the study design.

### Data Collection

**Figure 2:**
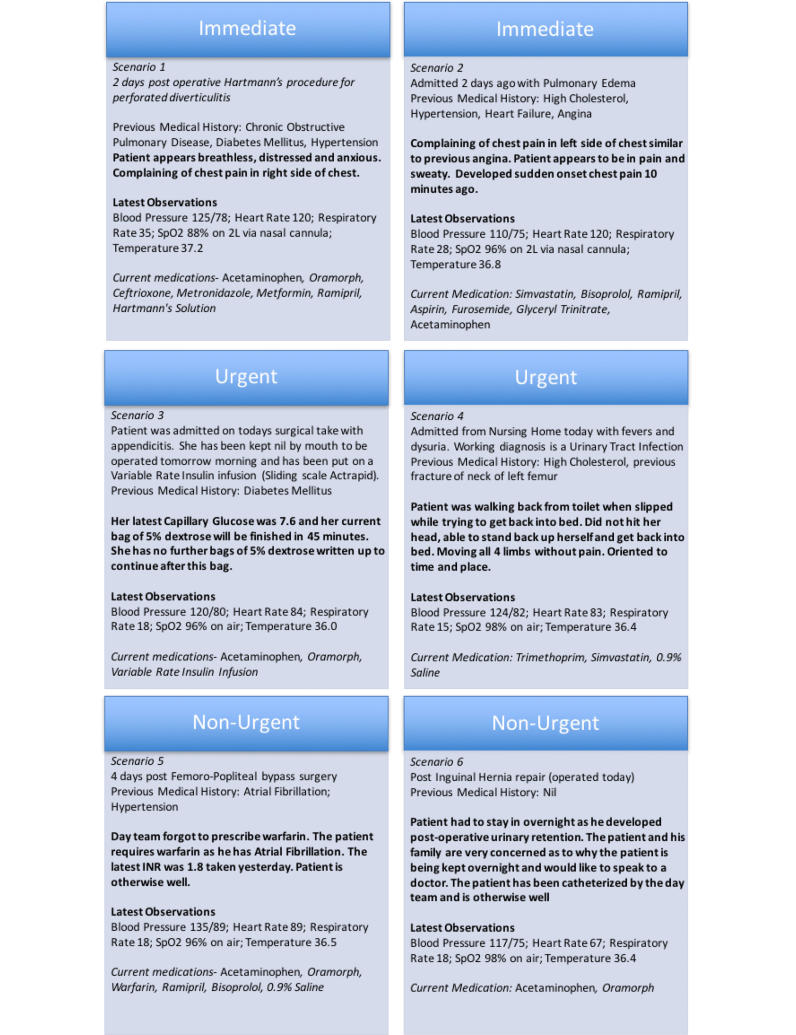
Outline of clinical scenarios. SpO_2_: oxygen saturation.

Participants were requested to use either a pager or Hark to give or receive information about simulated patients. We produced 6 scenarios, each describing a patient who required input from a doctor. In order to allow both urgent escalation of care and routine task management functions to be investigated, the scenarios varied in terms of the how quickly they required a response. In the 6 scenarios, 2 patients required immediate attention, 2 required urgent attention, and 2 required routine attention. All participates sent or received information about all 6 scenarios. Figure 2 details the scenarios.

In clinical practice referrals are usually made from nurses to doctors [8]; therefore, we presented nurse participants with the above scenarios and asked them to refer the patient to a member of the research team, who acted as a doctor. Referrals were made using either a paging device or Hark.

We asked doctor participants to carry a device, either a pager or a mobile phone with Hark installed, during a typical day at work. A member of the research team then randomly contacted them through the device to refer the patients from the scenarios. Doctors were able to differentiate messages sent for simulated patients from those about actual patients, as all pages were sent from a single telephone number reserved for the purposes of the study. All messages sent through Hark were about simulated patients.

These tasks were designed to model the day-to-day use of a pager device for doctors and nurses. Each participant was asked to perform the tasks on 2 separate occasions: once using a pager device and another time using Hark.

We collected data from observation and monitoring of participants during the task and from feedback questionnaires given to participants after the task was completed.

### Features of Hark

A full overview of the Hark platform and product overview can be seen at the Hark website [33]. Individuals register with Hark, creating a user profile from which they are able to send or receive messages. Their profiles contain information about their clinical role, experience level, and contact details.

To assign a task to colleagues, senders first select a patient and then enter details about the task required into a “new task” form in the app. As Hark integrates with the hospital’s electronic medical records, the form is prepopulated with the selected patient’s demographic information, recent vital signs, and test results. Users complete the remaining fields by selecting the type of task required and filling in free-text spaces with details about the task. At the end of the form, the sender selects a colleague to whom to send the task, indicating the urgency of the task by selecting a time frame for completion.

Once the form is sent, the colleague to whom the task was assigned receives a notification that a task has been sent to him or her. As soon as the recipient opens the form in the app, a notification is generated on the requesting device that the task has been read. The receiver can choose to accept the task, delegate it to another colleague, or send comments to clarify any further questions about the task. Once the task is completed it is marked as complete on the devices of both the sender and the receiver. A full audit trail of all communication episodes is made providing a contemporaneous record of all events associated with a clinical task.

### Measures

We created specific measures to allow the aims of the project to be achieved. To determine whether the quality of referrals differed between the devices, we audio-recorded the referrals made by nurses using the pager while the data from referrals made by using Hark were automatically recorded on the app itself. We then assessed the data against a previously validated assessment tool called the QUality of Information Transfer Tool (QUIT) [26]. The QUIT is a 25-item, 7-category assessment tool comprising core components to measure the quality and content of information transfer. To ensure consistency when using the QUIT, 2 independent researchers rated each referral.

The time taken for doctors to respond to referrals was recorded to determine whether using a new technology for escalation of care results in additional delays in communication. For the pager, we defined this as the time between when a page was sent and when a call was received in response to the page. For Hark, we defined this as the time between when a task was sent and when the device received a “message read” notification. The message read notification was deemed appropriate as a response, as this was the point (when using Hark) where information has been transferred from the sender to the receiver. By reading the message, the receiver can decide how quickly he or she needs to reply on the basis of that information. For nonurgent task requests, it would be appropriate for them to prioritize completing an urgent task in front of them above immediately replying on Hark. Furthermore, the message read notification provides assurance to the sender that the information has been viewed and appropriate action will be taken.

As referrals are usually made from nurses to doctors, we asked only nurses to make referrals, and therefore we used the QUIT only for nurse participants. Similarly, as we asked only doctors to respond to referrals, we collected data about the time taken to respond only for doctor participants.

Finally, to determine how end users perceive mobile phone apps as an alternative means of communication to pagers, we asked all participants to complete a questionnaire. The questionnaire asked participants to express their degree of agreement with 18 statements on a 5-point Likert scale. The statements correlated with the key requirements of a communication system that emerged from previously published research [11]. We sought trust ethical approval from the Imperial Joint Research Compliance Office, but because it was a simulated study that didn’t involve real patients, project approval alone was advised to be sufficient.

### Data Analysis

We analyzed data using SPSS statistics version 22 (IBM Corporation). We conducted descriptive analysis of the sociodemographic information.

Statistical analysis was performed to assess whether any significant differences existed in the performance of either device according to the QUIT scores, response times, and participants’ feedback. Aside from the time taken to respond to messages, the data were not normally distributed; therefore, we report median values and used the Wilcoxon test for within-group analysis. For the time taken to respond, we report mean values and used the paired-samples *t* test for within-group analysis. Spearman correlation coefficient was used to assess whether there was any relationship between the participants’ age and their evaluation of the device.

Statistical significance was accepted at a level of *P*<.05.

## Results

### Participants and Demographics

We included a total of 22 participants in the study, of whom 9 were doctors and 13 were nurses. All participants used both Hark and the pager device, which gave a total of 44 data points.

All doctors who participated were junior doctors (postgraduate year 1 and 2) with a mean age of 25 (range 24–28) years. Of the 9 doctor participants, 7 were female and 2 were male.

The seniority of nurses ranged from licensed practical nurses (pay scale band 5 in the United Kingdom) to advanced practice registered nurses (band 8) with a mean age of 38 (range 25–55) years. Of the 13 nurse participants, 7 were female and 6 were male.

The sample included representatives from medical and surgical specialties, along with psychiatry and pediatrics.

### Statistical Analyses

#### Quality of Information Transfer

**Figure 3:**
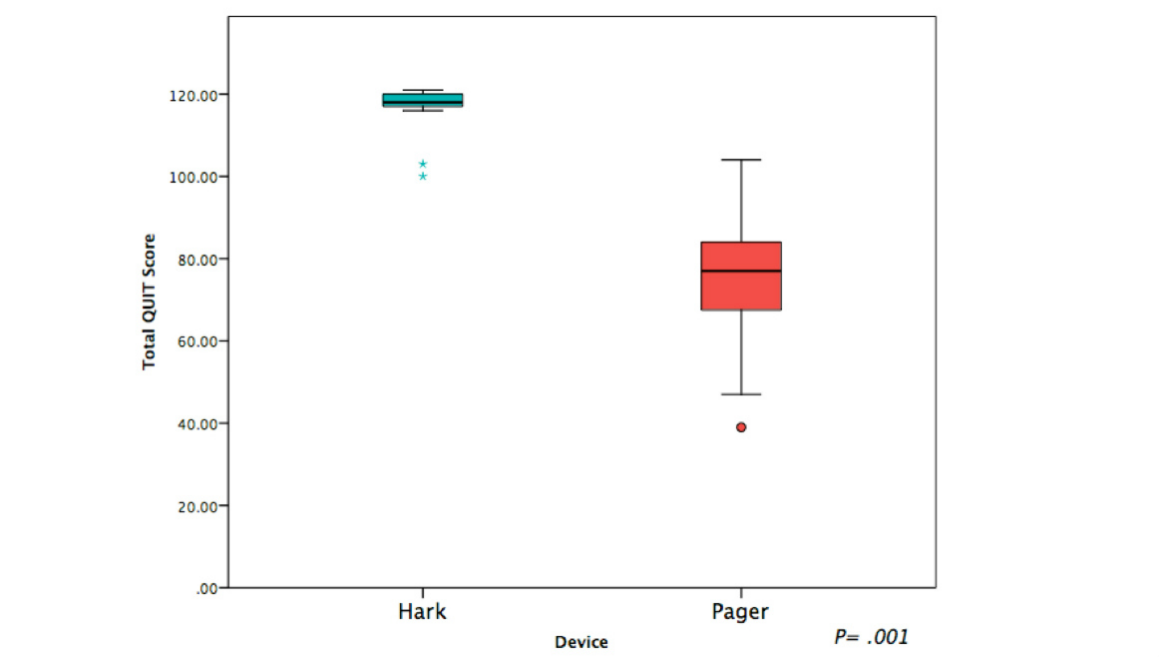
Box plots of overall quality of information transfer (QUIT) scores between devices. Asterisks and dot represent outliers.

The quality of the referrals made by nurses was significantly better when using Hark, with higher overall scores when using Hark than when using a pager device (Hark median 118, range 100–121 versus pager median 77, range 39–104; *P*=.001) (Figure 3). For all 7 categories, nurses were found to perform significantly better when using Hark (*P*<.05). Furthermore, 22 of the 25 QUIT items were conveyed significantly more frequently when using Hark. There was no significant difference in the frequency of communicating the “patient name,” “patient location,” “responsible consultant [clinician],” and “current treatment to date.” These results provide good evidence that Hark improves the quality of referrals (see Table 1).

#### Time Taken to Respond to Device

Doctors responded to messages using Hark more quickly than when responding to pagers, although this difference was not statistically significant (see Table 2). There was also no statistically significant difference when comparing response times to messages requiring immediate, urgent, and nonurgent response.

#### Evaluation of the Device

Hark was found to perform significantly better on 11 of the 18 criteria of an information transfer device (*P*<.05; Table 3). These included “enhances interprofessional efficiency,” “results in less disturbance,” “performed desired functions reliably,” and “allows me to clearly transfer information.” On the remaining 7 items, users rated Hark as better than or equal to the pager, but the difference was not statistically significant. Users also reported a greater overall satisfaction when using Hark, although this difference was also not significant (Hark median 4, range 1–5 versus pager median 3.5, range 1–5; *P*=.24).

Older users were more likely to rate the pager highly on enabling them “to both send and receive communications” (correlation coefficient 0.538 *P*=.01). Age did not correlate with ratings of either device on any other criteria (Table 4).

**Table 1:** Quality of information transfer scores for use of Hark versus a pager.

Categories and items		Hark		Pager		*P* value
		Median	Range	Median	Range	
**1) Communicator identities**		15	11–15	7	3–13	.001
	Clearly communicates initiator identity (include grade and specialty)	5	5–5	4	1–5	.004
	Clearly confirms appropriate receiver identity (include grade and specialty)	5	5–5	2	1–4	.001
	Establishes rapport and mutual respect	5	1–5	3	1–5	.004
**2) Patient identity**		16	11–18	11	7–17	.003
	Clearly communicates patient name	5	4–5	4	1–5	.03
	Clearly communicates patient location	5	1–5	5	2–5	.33
	Clearly communicates responsible consultant	1	1–3	1	1–5	.91
	Clearly communicates age/date of birth	5	4–5	1	1–5	.002
**3) Clinical details**		14	9–15	11	3–14	.003
	Clearly articulates working diagnosis	5	3–5	4	1–5	.009
	Clearly communicates relevant history (including recent operation and date)	5	1–5	4	1–5	.004
	Outlines current treatment to date	5	2–5	3	1–5	.03
**4) Problem**		30	25–30	18	11–28	.001
	Clearly describes current problem with patient	5	5–5	4	3–5	.01
	Communicates relevant vital signs and fluid balance	5	3–5	4	2–5	.004
	Describes patient assessment and examination findings	5	2–5	3	1–4	.002
	Outlines relevant investigation results to date	5	3–5	3	1–4	.001
	Effectively prioritizes clinical issues	5	5–5	2	1–5	.002
	Clearly communicates degree of urgency	5	5–5	2	1–5	.003
**5) Plan**		15	12–15	11	5–14	.002
	Clearly defines the reason for the call (eg, advice, patient review, transfer)	5	3–5	4	2–5	.002
	Definitively resolves questions and ambiguities about patient care	5	4–5	3	1–4	.002
	Agrees plan for ongoing care for patient	5	3–5	4	1–5	.02
**6) Overall quality of information presentation**		30	17–30	18	8–29	.001
	Uses clear, understandable language throughout	5	5–5	4	2–5	.002
	Presents information in a structured and logical order	5	5–5	3	1–5	.002
	Used available documentation to structure handoff	5	3–5	5	1–5	.003
	Selected and communicated all relevant information	5	5–5	3	1–5	.002
	Completed information transfer without digressing	5	5–5	3	1–5	.002
	Overall quality of information transfer	5	4–5	3	1–4	.002
Total score		118	100–121	77	39–104	.001

**Table 2:** Time taken to respond to various types of messages transmitted by Hark versus a pager.

Type of message	Hark			Pager			*P* value
	Mean	SD	Range	Mean	SD	Range	
All messages, time in seconds	86.6	96.2	2–416	136.5	201.0	4–900	.11
Immediate messages, time in seconds	126.0	128.4	2–416	80.6	81.2	4–300	.22
Urgent messages, time in seconds	68.1	84.3	4–286	130.2	168.0	9–585	.12
Nonurgent messages, time in seconds	65.8	52.9	8–195	198.8	289.4	15–900	.07

**Table 3:** Performance of Hark versus a pager as evaluated by agreement on a 5-point Likert scale with criteria for an information transfer device.

Statement	Hark		Pager		*P* value
	Median	Range	Median	Range	
I do not need to be in a specific location within the hospital to initiate or receive communication through this system	5	1–5	5	1–5	.15
I am able to send or receive sufficient levels of detail through this system	5	1–5	4	1–5	.31
It enhances interprofessional collaboration and efficiency	4	1–5	3	2–5	.01
It results in fewer interruptions	5	2–5	2	1–5	.001
It results in less disturbance from interruptions	4	2–5	2	1–5	.003
It minimizes the time between sending a message and receiving the desired response	4	1–5	3	1–5	.22
It makes it easy to contact colleagues in times of need	4	1–5	3.5	2–5	.37
It discourages transfer of unnecessary information	4	1–5	3	1–5	.07
It is simple to operate	4	2–5	4	2–5	.75
It allows me to both send and receive communication	5	1–5	4	1–5	.003
It allows me to clearly transfer information about tasks and patients	5	1–5	4	1–5	.01
It allows me to easily delegate tasks or patients to colleagues	5	3–5	3	1–5	.001
It allows me to access patient information	4	2–5	2	1–5	.001
It allows me to prioritize messages according to urgency	5	1–5	2.5	1–5	.002
It performs the desired functions reliably, with minimal occurrence of malfunctions	4	2–5	3.5	1–5	.01
It can be stored as evidence that communication occurred	5	1–5	2	1–5	.001
It can allow a third person to differentiate between different senders and receivers	5	1–5	2	1–5	.001
I would be satisfied if this was the primary system used for communication between wards staff and doctors	4	1–5	3.5	1–5	.24

**Table 4:** Correlation between age of participants and their rating of a device by agreement with evaluation criteria.

Statement	Hark		Pager	
	Spearman correlation coefficient	*P* value	Spearman correlation coefficient	*P* value
I do not need to be in a specific location within the hospital to initiate or receive communication through this system	–.160	.48	–.125	.58
I am able to send or receive sufficient levels of detail through this system	–.162	.47	.155	.49
It enhances interprofessional collaboration and efficiency	.118	.60	.196	.38
It results in fewer interruptions	–.175	.44	.381	.08
It results in less disturbance from interruptions	–.203	.37	.417	.05
It minimizes the time between sending a message and receiving the desired response	.032	.89	.216	.34
It makes it easy contact colleagues in times of need	.100	.66	–.260	.24
It discourages transfer of unnecessary information	.096	.67	.400	.07
It is simple to operate	–.051	.82	.172	.45
It allows me to both send and receive communication	–.301	.17	.538	.01
It allows me to clearly transfer information about tasks and patients	–.076	.74	.041	.86
It allows me to easily delegate tasks or patients to colleagues	–.013	.96	.417	.05
It allows me to access patient information	–.087	.70	.287	.20
It allows me to prioritize messages according to urgency	.066	.77	.075	.74
It performs the desired functions reliably, with minimal occurrence of malfunctions	–.147	.51	.254	.26
It can be stored as evidence that communication occurred	–.376	.09	–.394	.07
It can allow a third person to differentiate between different senders and receivers	–.405	.06	.262	.24
I would be satisfied if this was the primary system used for communication between wards staff and doctors	.035	.88	.271	.22

## Discussion

Our study aimed to investigate the efficiency of a user-centered ABCS compared with a pager device for interprofessional communication in hospitals. It compared the quality of information transfer during a patient referral, the time taken for users to respond to referrals, and user perceptions of interprofessional communication using mobile phone apps as an alternative means of communication to pagers.

**Figure 4:**
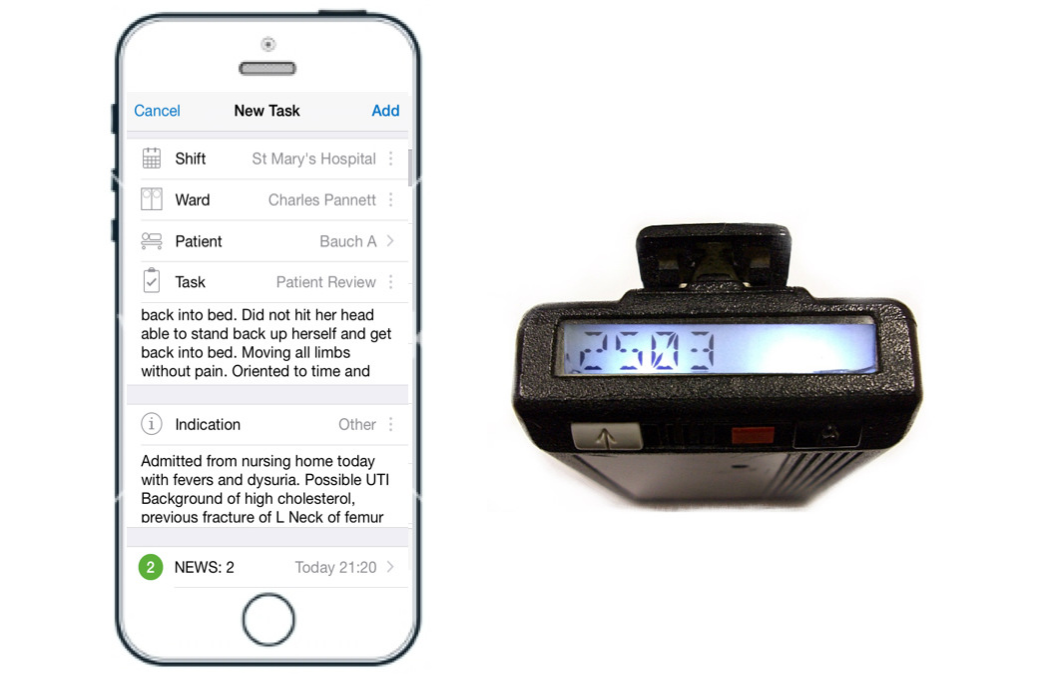
Information about a simulated patient displayed in messages sent over Hark and a pager.

We found the quality of information transfer to be higher when using the Hark app than when using the pager. Further analysis of the results revealed that. aside from 3 parameters (patient name, location, and responsible clinician), all relevant pieces of information were transferred significantly more frequently when using Hark than when using a pager. To transfer information using Hark, users must complete several fields, each of which corresponds to a piece of information that needs to be transferred. As such, the task entry form is a template. with each field serving as a prompt to ensure more complete information transfer [34]. This is in contrast to the pager, where the only information transferred is a number to be called back (see Figure 4).

There was no field for entering the name of the responsible clinician, which may explain the equal frequency of information transfer of this parameter when using Hark or the pager. The other 2 parameters that we found to be nonsignificant (patient name and location) were so essential and intuitive for a referral that prompting should not be necessary to ensure transfer of the information.

The data demonstrated no significant difference in the time taken to respond to messages sent through either device. Is it noteworthy that, despite using a newer device with little training and limited prior experience of it, users did not take longer to respond to referrals sent using Hark. It should be noted that the time taken to respond to messages was defined differently according to the device used. When using Hark, response time was defined as the time taken to receive a message read notification. When using the pager, this was defined as the time taken for the receiver to return the call. This may introduce some bias in favor of Hark; however, this is a reflection of how reading a message through Hark results in earlier transfer of information. This suggests that in a clinical setting using Hark would not result in any additional delay over using a pager when contacting colleagues for escalation of care purposes. Indeed, Joseph et al found that at a hospital that used mobile phones in the clinical environment, users reported a reduction in physician response time to both routine and critical patients compared with when using pagers [23].

Users of Hark reported that it performed significantly better than the pager on most of the parameters we assessed. Specifically, they perceived Hark to be significantly better at enhancing interprofessional collaboration, reducing disruption, enabling task prioritization, and improving reliability. There were no instances in which users reported better performance when using the pager. This suggests that the participants of this study would support switching the devices used on wards from pagers to app-based systems [35]. These findings support the conclusions of other studies in the literature that explore altnernatives to the pager. Interventions such as 2-way alphanumeric pagers, task management systems, and secure text messaging platforms all result in users reporting improved efficiency in communication and reduced disruption [14,15,36].

Using an ABCS offers several benefits for patients, health care professionals, and managers alike. As we have shown, using Hark results in a higher quality of information transfer, which results in clearer communication and may lead to improved patient care. Using Hark allows health care professionals to communicate more effectively, resulting in greater interprofessional collaboration and less disruption to their work. As all communication is stored and indexed according to the type of tasks requested, Hark offers managers fresh insights about the most frequent types of tasks performed in their hospitals and thus supports decision making about resource workforce management. The audit trail provided by Hark would also facilitate incident investigation and keeping staff portfolios and logbooks. It is important to be aware of possible unintended consequences when using an ABCS. As more information is transferred over the technology, the number of verbal and face-to-face communications may decrease, negatively affecting workplace relationships. Furthermore, complex situations may be oversimplified when communicated over an ABCS, resulting in greater back-and-forth messaging for clarification [37].

While there are many demonstrable benefits of using an ABCS, some essential pieces of infrastructure need to be in place before it can be widely adopted in the health care setting. First, there must be secure Internet or mobile phone coverage throughout the hospital to allow users to be able to send and receive information regardless of their location. This is particularly important if time-critical information regarding deteriorating patients is to be sent over Hark, as delays in escalation of care are associated with adverse outcomes [25]. Second, as the ABCS requires a mobile phone for use, all health care workers must have access to a mobile phone or tablet device. Although most health care workers already carry personal mobile phones, provision of mobile phones or tablets should be considered for staff who do not have access to a device [38]. Third, the app must be constantly running on the mobile phone device, which will challenge the battery life of most popular devices. As such, hospitals that use Hark must ensure widespread access to mobile phone charging facilities.

### Limitations

This study has certain limitations, which future research should seek to address. First, this was a single-center study in a simulated setting. Hark should be tested across other sites in different geographic areas to determine whether the results obtained in this study can be replicated. As the study used 6 simulated cases as opposed real patient situations, participants may have evaluated the devices differently from how they would have if we had used in real clinical scenarios. In particular, users may have found Hark to be less disruptive when using these 6 scenarios than they would have when considering more cases with real clinical care.

Doctors’ awareness before responding that all cases were about simulated patients may have influenced how quickly they responded to messages during our study. They may have deprioritized their response to messages about simulated patients or may have responded more quickly when using a preferred technology. To minimize this preference bias, we did not tell participants that we would be monitoring their response time. Future studies could aim to minimize this bias further by blinding participants between messages about simulated and actual patients.

It should also be noted that the sample of doctors who participated in this study consisted only of junior doctors. Although our results did not demonstrate a relationship between age and evaluation of the devices used, future research should include all grades of doctor to minimize any potential bias introduced by the age of the participant. It should also be noted that, although the study had a limited sample size of 22, the crossover design of the study allowed a larger number of data points to be collected from a smaller number of participants. The sample size was therefore sufficient to provide the required number of data points. The crossover design provided the additional benefit of minimizing bias between groups.

The implication of this study is that teams using Hark in clinical practice can transfer information more effectively so that clinicians can potentially prioritize their patients using objective, physiological parameters. This may result in fewer treatment delays and prevent avoidable harm [39-41]. Future research should aim to assess how the ABCS performs in a clinical environment when in use as a principal modality of communication and should include analysis of whether use of Hark has an impact on error rates and avoidable adverse events. Using the ABCS first in a pilot ward and then expanding from there may facilitate these efforts.

### Conclusions

This study has investigated an alternative to the pager system for information transfer and task management. Hark has been demonstrated to improve the quality of information transfer and has been rated by users as more effective on several important measures, without any reduction in user satisfaction in a simulated environment. Using this device resulted in no delay in patient care. As one of the vital components of safe clinical care, improving interprofessional communication is a priority, and systems such as Hark can support this aim.

## Abbreviations

ABCS: app-based communication system
QUIT: QUality of Information Transfer Tool
SMS: short message service
